# Neutrophil activation during attacks in patients with hereditary angioedema due to C1-inhibitor deficiency

**DOI:** 10.1186/s13023-015-0374-y

**Published:** 2015-12-10

**Authors:** Nóra Veszeli, Dorottya Csuka, Zsuzsanna Zotter, Éva Imreh, Mihály Józsi, Szabolcs Benedek, Lilian Varga, Henriette Farkas

**Affiliations:** Hungarian Angioedema Center, 3rd Department of Internal Medicine, Semmelweis University, Kútvölgyi út 4, H-1125 Budapest, Hungary; Urology Department, Medical Center, Hungarian Defence Forces, Budapest, Hungary; Central Laboratory, Kútvölgyi Clinical Block, Budapest, Hungary; MTA-ELTE “Lendület” Complement Research Group, Department of Immunology, Eötvös Loránd University, Budapest, Hungary; Haematology Unit, 3rd Department of Internal Medicine, Semmelweis University, Budapest, Hungary

**Keywords:** Hereditary angioedema, C1-inhibitor deficiency, Edematous attack, Neutrophil granulocytes, Neutrophil activation, Neutrophil elastase, Myeloperoxidase, IL-8, TNF-α

## Abstract

**Background:**

Earlier studies have shown that the absolute number of neutrophil granulocytes (NGs) may increase during attack of hereditary angioedema due to C1-inhibitor deficiency (C1-INH-HAE). Whether NGs undergo activation during attack has not yet been investigated. However, as neutrophil elastase (NE) can cleave and inactivate C1-INH which may contribute to the dysregulation of the kallikrein-kinin system and hence, to edema formation. Our aim was to investigate the possible activation of NGs during attacks.

**Methods:**

We studied blood samples obtained from 26 patients with C1-INH-HAE during symptom-free periods and during attacks, along with samples from 26 healthy volunteers. NG count (NGC), NE, myeloperoxidase (MPO), pentraxin 3 (PTX3), CRP, C5a, factor H, IL-8, and TNF-α levels were measured.

**Results:**

NGC was higher during attacks than during symptom-free periods (*p* = 0.0132), and the same was observed for NE (*p* = 0.0026), MPO (*p* = 0.0008), and PTX3 levels (*p* = 0.0409). There was a strong positive correlation between NE and MPO levels during attacks (*p* < 0.0001, *R* = 0.709). Furthermore, IL-8 (*p* = 0.0061) and TNF-α (*p* = 0.0186) levels were also elevated during attacks, compared with symptom-free periods. By contrast, C5a and factor H levels were similar in samples obtained during attacks or in symptom-free periods.

**Conclusion:**

Increased NGC was associated with elevated NE and MPO levels – this suggests neutrophil activation during attacks. The strong positive correlation between NE and MPO levels, together with the elevated PTX3 concentration, may indicate the expression of neutrophil extracellular traps. All these processes may contribute to the activation of kallikrein-kinin system, which leads to the onset of an edematous episode.

## Background

Hereditary angioedema due to C1-inhibitor deficiency (C1-INH-HAE) is a rare, autosomal dominant disorder. It is caused by the reduced antigenic level and/or functional activity of the C1-inhibitor (C1-INH), resulting from a mutation in the gene encoding C1-INH *(SERPING1)* [1].

The serine protease inhibitor C1-INH is the primary regulator of the classical and lectin complement pathways—and also of the kallikrein-kinin, coagulation and fibrinolytic systems [2]. In the deficiency of C1-INH, the kallikrein-kinin system undergoes activation and this result in the cleavage of bradykinin from high-molecular-weight kininogen (HK)—a process catalyzed by kallikrein. Bradykinin increases vascular permeability and thereby induces the extravasation of plasma into the tissues, leading to edema-formation [3].

C1-INH-HAE is characterized by recurring episodes of subcutaneous and/or submucosal edema [4]. Acute edema formation in the upper airways may even cause asphyxia from airway obstruction [5]. As the determinants of the immediate cause, the time of onset, or the location of edema formation remain unknown, the occurrence of these episodes cannot be predicted in advance. Although much progress has been made during recent years in exploring the pathophysiology of the disease, research has largely focused on the role of the different plasma enzyme systems [3, 6].

Previously, a number of case studies reported the elevation of white blood cell (WBC) count and neutrophil granulocyte counts (NGC) during edematous attacks [7–10]. Some authors attributed this to hemoconcentration from the extravasation of plasma during the edematous episode [7, 8]. In 2010, our team confirmed these reports in a study conducted on 18 HAE patients: we found increased WBC count and NGC during edematous episodes. Further, we showed that the increase of NGC during the attack was greater than could be explained by hemoconcentration [11].

**Fig. 1:**
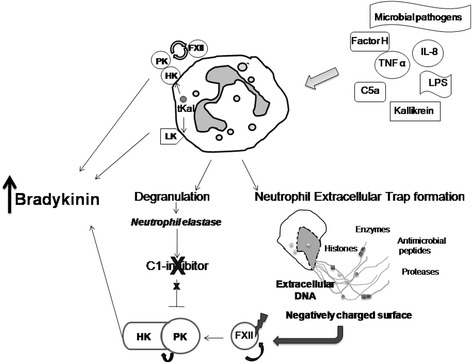
Activation of neutrophil granulocytes and the kallikrein-kinin system. During neutrophil activation triggered by different substances, the released neutrophil elastase could cleave and inactivate C1-INH [12]. Besides, activated neutrophils can release neutrophil extracellular traps, and both processes may contribute to bradykinin release [13, 15]. On the other hand, high molecular weight kininogen and factor XII can attach directly to the surface of NGs. Prekallikrein, by contrast, binds to the cell membrane indirectly, through its docking protein, high molecular weight kininogen, which could create the conditions for the release of kinins (bradykinin and kallidin) through the activation of the cell-bound kallikrein-kinin system. This would be manifested by the factor XII-mediated activation of prekallikrein on one hand, and/or by the release of neutrophil-borne, active tissue kallikrein on the other [17]. [Abbreviations: IL = interleukin, TNF-α = tumor necrosis factor-α, LPS = lipopolysacharide, HK = high molecular weight kininogen, PK = prekallikrein, tKal = tissue kallikrein, LK = low molecular weight kininogen, C1-INH = C1-inhibitor, FXII = factor XII, MPO = myeloperoxidase, PTX3 = pentraxin 3]

Notwithstanding these findings, the possible activation of NGs in HAE attacks has not yet been investigated. This is all the more strange, since NGs are known to have the potential to exert multiple influences on the kallikrein-kinin system. Neutrophil elastase (NE) –released from activated NGs- can cleave and inactivate C1-INH [12]. This may contribute to the dysregulation of plasma enzyme systems and hence, to edema formation, as C1-inhibitor is the most potent regulator of the kallikrein-kinin system, by controlling the activity of kallikrein and of activated factor XII [2]. The activation of NGs may lead to the formation of neutrophil extracellular traps (NETs), which are filamentous structures of DNA and histones containing granular enzymes [NE and myeloperoxidase (MPO), primarily] along with antimicrobial peptides [defensins, and pentraxin 3 (PTX3)] [13, 14]. NETs can provide a negatively charged surface suitable for the activation of the kallikrein-kinin and of the complement systems [15, 16]. On the other hand, the kallikrein-kinin system can be activated also on the surface of neutrophils [17] (Fig. 1).

A variety of factors related to the activation of NGs have been identified [18–20], and all these might have their role in the pathomechanism of edema formation.

The objectives of our study were as follows:

1. To confirm the previously described increase of NGC in a larger patient population, by analyzing peripheral blood samples obtained from the same C1-INH-HAE patients during symptomatic and symptom-free periods.
2. To investigate the possible activation of NGs during edematous episodes, by determining the levels of the enzymes NE and MPO (both released from primary granules during activation), as well as of the protein PTX3 (released from secondary granules) in peripheral blood samples [20].
3. To measure the levels of interleukin-8 (IL-8) and tumornecrosis factor-α (TNF-α) (the cytokines with the greatest influence on neutrophil activation), of C5a (a complement anaphylatoxin, which induces neutrophil activation), and of factor H (involved in the regulation of neutrophil activation), as well as of C-reactive protein (CRP) in peripheral blood samples [19, 20].

## Methods

### Patients

The subjects were selected from the patient population (*n* = 172) receiving follow-up care at the Hungarian Angioedema Center. We included 26 patients (20 females, 6 males, mean age: 35.8 years) who presented at the Center for treatment for an acute edematous episode; 19 patients had type I, whereas 7 had type II C1-INH-HAE. The diagnosis of C1-INH-HAE was established by pedigree-analysis, as well as by the evaluation of the clinical manifestations and the complement parameters (C1-INH antigenic and functional levels, C1q, C4). Nine patients were on continuous danazol treatment, whereas the remaining 17 did not receive long-term prophylaxis. Human plasma-derived C1-INH concentrate (Berinert®, CSL Behring, Marburg, Germany) was reserved for the acute treatment of edematous attacks. The “symptom-free samples” were collected during the annual control visits. None of the patients had any clinical manifestation suggestive of an acute infection during the edematous attack and during annual control visits.

### Healthy controls

The control group consisted of 26 healthy adults (19 females and 7 males, mean age: 35.2 years). All had been referred for routine medical examination. The healthy controls did not have any known disease, and did not receive medicinal products at the time of blood sampling. C1-INH deficiency was excluded by complement testing (measuring antigenic and functional level of C1-INH, C4, and C1q).

The C1-INH-HAE patients and the controls were not statistically different as regards age and gender distribution.

### Blood sampling

Peripheral blood samples were obtained from patients with C1-INH-HAE both during symptom-free periods and during attacks (before acute treatment) as well as from healthy subjects. EDTA plasma and serum samples were stored at −70 °C until processing.

The study protocol was approved by the institutional review board of Semmelweis University of Budapest, and informed consent was obtained from the participants in accordance with the Declaration of Helsinki.

### Measurement of the parameters related to neutrophil activation and to the complement parameters of C1-INH-HAE

WBC count, NGC, Red blood cell (RBC) count and hematocrit were determined in the samples using Advia 120 Hematology System automate (Siemens, Erlangen, Germany). A stable complex of NE with alpha1-proteinase-inhibitor, and MPO were measured in EDTA-plasma by sandwich type ELISA (QIA96, Calbiochem, Merck-Millipore, Darmstadt, Germany, and Immundiagnostik AG, Bensheim, Germany). For the measurement of PTX3 level, we used a Duoset ELISA kit (R&D systems, Minneapolis, USA). Commercially available high-sensitivity ELISA kits were used to measure the levels of cytokines (IL-8, TNF-α—R&D system, Minneapolis, USA and Thermofisher Scientific Inc, Waltham, USA), and of the C5a anaphylatoxin (Quidel, San Diego, USA) in EDTA plasma. All procedures were carried out according to the manufacturer’s instructions. Serum levels of CRP were determined using a chemistry analyzer (Beckman Coulter Inc., California, USA).

The concentration of factor H was determined with an in-house sandwich ELISA method. In brief, 96-well ELISA plates (Nunc, Denmark) were coated with 1:1000 dilution of sheep anti-human factor H IgG fraction (The Binding Site Inc., Birmingham, UK), and incubated overnight at 4 °C. The next day, the plates were blocked for 1 hour using PBS with 0.5 % BSA and then, incubated containing serum samples diluted 1000-fold. A mixture of sera from healthy individuals (concentration = 557 μg/ml, calibrated with recombinant factor H) was used as reference standard after a seven-step series of two-fold dilutions, starting from a dilution ratio of 1:250. The samples and the standard were diluted with PBS-Tween containing 0.5 % BSA. After an 1-hour incubation, mouse monoclonal anti-human factor H (Quidel, San Diego, USA) was dispensed to the plates in 1:2000 dilution. Following incubation for an additional hour, goat anti-mouse IgG-HRP (Southerm Biotech, Birmingham, Alabama, USA) was added in 1:8000 dilution. 1,2-phenylenediamine dihydrochloride (OPD, DAKO Denmark A/S, Glostrup, Denmark) was used as substrate; the color reaction was stopped by the addition of 0.4 N sulphuric acid and optical density was measured at 492 and 620 nm wave lengths.

Radial immunodiffusion was performed to measure C4 level (polyclonal rabbit anti-human C4c Complement, DAKO Denmark A/S, Glostrup, Denmark), as well as the concentration of antigenic C1-INH (goat antisera to human C1-INH, Quidel, San Diego, CA, USA). Functional C1-INH level was determined with a commercially available ELISA kit (Quidel, San Diego, CA, USA), according to the manufacturer’s recommendations.

All analyzed parameters were determined in the same, previously unthawed aliquot from each subject.

### Statistical analysis

Statistical analysis was performed with Prism for Windows 5.0 (Graph-Pad Software, San Diego, CA, USA) statistical software. As many of the variables had non-Gaussian distributions, we used non-parametric tests throughout the analysis. Mann–Whitney’s U-test was applied to compare two independent groups (C1-INH-HAE-patients *vs.* healthy controls), whereas the Wilcoxon test (a paired t-test) was chosen to compare “symptom-free” and “during attack” values from the same patients. Correlations were calculated with the Spearman’s rho test. All the statistical analyses were two-tailed, and *p* < 0.05 was considered to represent a significant difference, or correlation.

## Results

### Analysis of neutrophil granulocyte counts

**Fig. 2:**
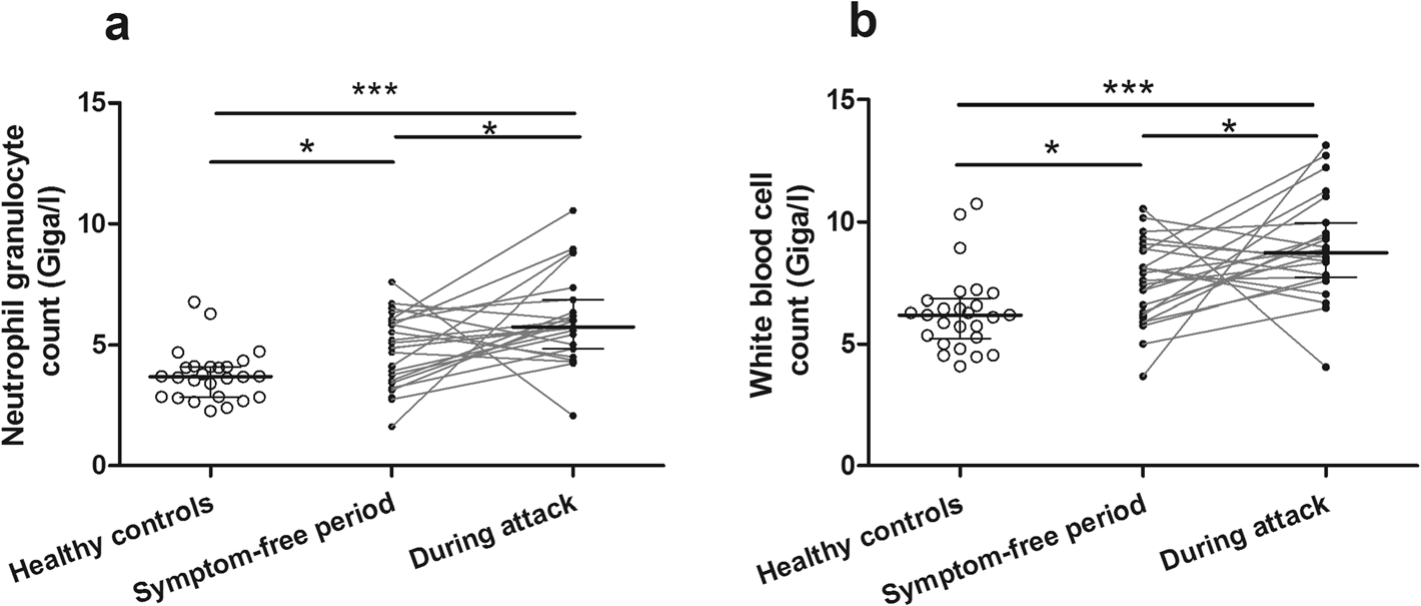
NGC^‡^ (**a**) and WBC^‡^ count (**b**) in blood samples drawn during attacks and in symptom-free periods from patients with C1-INH-HAE, and from healthy controls. Median and interquartile ranges are shown. (*p < 0.05, **p < 0.01, and ***p < 0.001; Wilcoxon signed-rank test and Mann-Whitney U test). ^‡^Absolute cell counts were corrected for hemoconcentration occurring during attacks

Because the extravasation of fluid into the extracellular space may result in hemoconcentration of variable extent, we adjusted “during attack” WBC and NGC values with the latter before making comparisons among the study groups. The magnitude of hemoconcentration was estimated in individual patients by taking into account the ratio of RBC counts determined during attacks and during symptom-free periods. The observed WBC count and NGC measured in during attack samples were divided by the calculated ratio (RBC count during attack/RBC count during symptom-free period) in each patient, to eliminate the changes induced by hemoconcentration. We made the comparative analysis with these “corrected” values. Comparing symptom-free C1-INH-HAE patients and controls, we found higher NGC in patients than in controls (median: 4.87 vs. 3.69 Giga/l; *p* = 0.0107, *Mann-Whitney test*). Subsequently, we confirmed this observation in another population of 114 C1-INH-HAE patients and 210 healthy subjects (*p* = 0.0002). Moreover, we found that compared with symptom-free periods, this difference increases further during edematous episodes (median: 4.87 vs. 5.74 Giga/l; *p* = 0.0132, *paired t-test*) in the same C1-INH-HAE patients. We observed similar differences among the three study groups with respect to WBC [symptom-free vs. healthy controls (median: 7.52 vs. 6.19 Giga/l; *p* = 0.0165) and symptom-free vs. during attack (median: 7.52 vs. 8.73 Giga/l; *p* = 0.0254)] (Fig. 2). When we made the corrections for hemoconcentration with the above mentioned methods, using hematocrit values of each patient, we observed the same significant differences between symptom-free and during attack samples of the patients (*p* = 0.0212 for NG and *p* = 0.0321 for WBC) as it was found when we made the corrections using RBC values.

### To probe for neutrophil activation

**Fig. 3:**
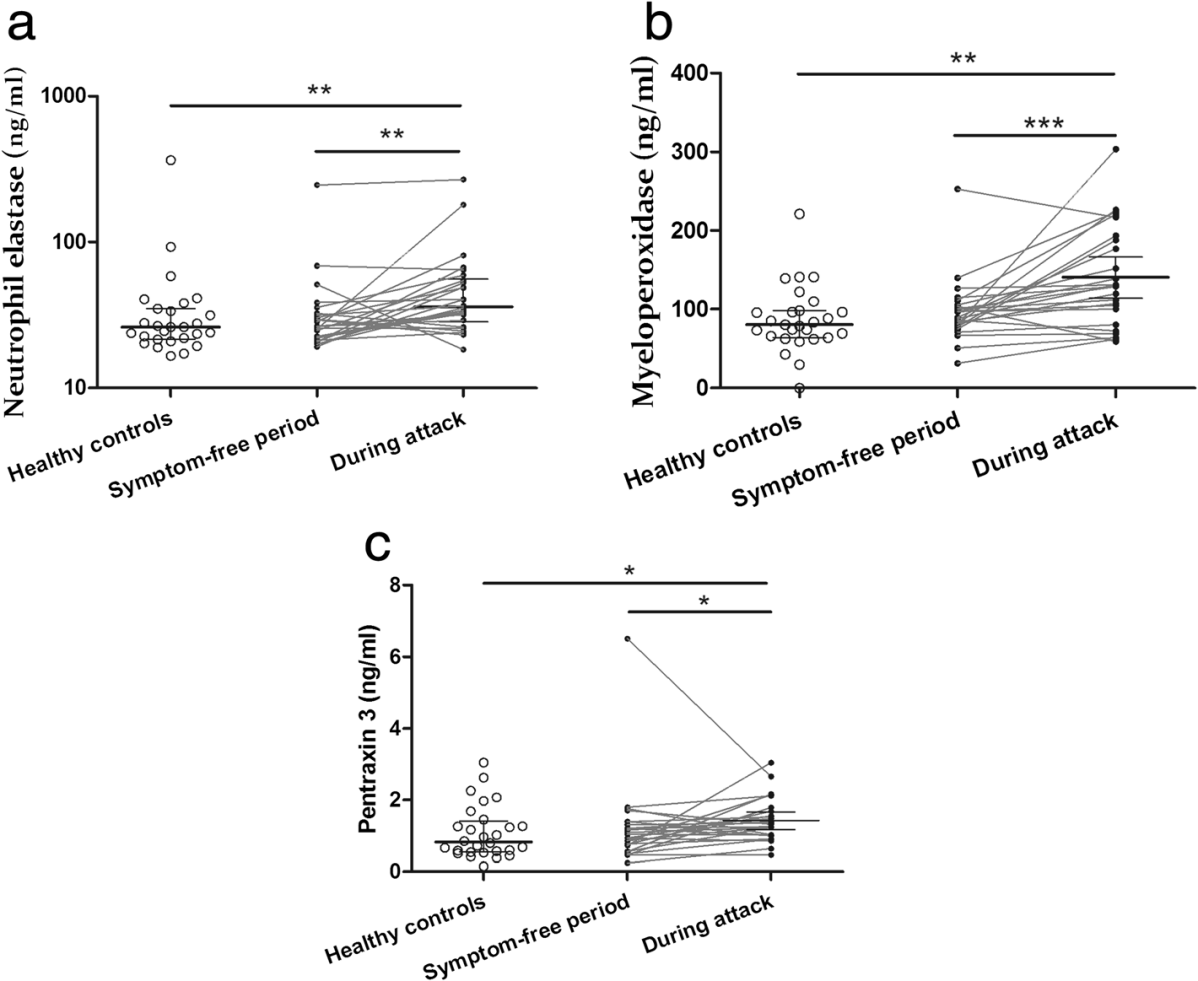
The levels of NE (**a**), MPO (**b**), and PTX3 (**c**) in blood samples drawn during attacks and in symptom-free periods from patients, compared with healthy controls. Median and interquartile ranges are shown. (*p < 0.05, **p < 0.01, and ***p < 0.001; Wilcoxon signed-rank test and Mann-Whitney U test)

In order to analyze neutrophil activation, we measured the levels of NE and of MPO, as well as of PTX3. In symptom-free patients, none of these differed from the corresponding values of healthy controls. However, the levels of all three markers were significantly higher in samples obtained during edematous attacks. The differences were statistically significant, compared both with symptom-free samples of the same patients (NE: 35.90 *vs.* 26.40 ng/ml; *p* = 0.0026, MPO: 129.0 *vs.* 89.40 ng/ml; *p* = 0.0008; and PTX3: 1.28 *vs.* 0.98 ng/ml; *p* = 0.0409), and with samples from healthy controls (NE: 35.90 *vs.* 26.07 ng/ml; *p* = 0.0043, MPO: 129.0 *vs.* 80.14 ng/ml; *p* = 0.0024; and PTX3: 1.28 *vs.* 0.82 ng/ml; *p* = 0.0157) (Fig. 3).

### Analysis of cytokines, CRP and complement factors

IL-8 levels were comparable in samples obtained from symptom-free patients and from healthy controls. During edematous attacks, however, IL-8 levels were higher than during symptom-free periods [median (25–75 percentile) 2.43(1.95–5.46) *vs*. 1.69 (0.82–2.71) pg/ml; *p* = 0.0061] in the same C1-INH-HAE patients. The situation was similar for TNF-α levels (1.27 (0.83–1.97) *vs.* 0.81 (0.04–1.64) pg/ml; *p* = 0.0186), with the distinction that these were significantly lower in symptom-free patients than in healthy controls (median: 0.81 (0.04–1.64) *vs.*1.78 (0.56–2.30) pg/ml; *p* = 0.0296). As the latter was an unexpected finding, we repeated TNF-α measurements in another set of samples from different C1-INH-HAE patients (*n* = 31) and healthy individuals (*n* = 57). This check confirmed the result of the above comparison, and showed significant differences between the TNF-α levels in samples from symptomatic or symptom-free patients (*p* = 0.0159), as well as in those from symptom-free patients or healthy controls (*p* = 0.0015).

Serum CRP level was higher during the symptom-free period, compared with that observed in the healthy subjects (median: 2.20 (1.85–3.78) *vs.* 1.50 (0.85–2.17) mg/l; *p* = 0.0031), and increased further significantly during attacks (median: 2.20 (1.85–3.78) *vs.* 3.39 (1.96–4.98) mg/l; *p* = 0.0321) in the same C1-INH-HAE patients.

There were no significant differences between the C5a and factor H levels measured in samples from the patients and from the controls (C5a: 9.61 (6.51–13.87) *vs.*10.45 (7.92–14.04) ng/ml; p = n.s. and factor H: 711 (527–878) *vs.* 596 (431–775) μg/ml p = n.s.). The same was found in samples obtained during attacks or in symptom-free periods (C5a: 10.51(8.57–15.63) vs. 9.61(6.51–13.87) ng/ml p = n.s.; factor H: 625 (533–711) vs. 711 (527–878) μg/ml; p = n.s.).

### Relationships among the parameters measured in patients with C1-INH-HAE and in healthy controls

Substantial neutrophil activation does not occur in healthy individuals and hence, a significant correlation between NGC and the levels of activation markers cannot be expected. The analysis of the mutual relationships among all parameters measured in healthy controls revealed only one negative correlation—that is, between the levels of TNF-α and of C5a (*R* = −0.4464, *p* = 0.0373). Further, we did not find a significant correlation among the parameters measured in the samples from symptom-free C1-INH-HAE patients.

**Fig. 4:**
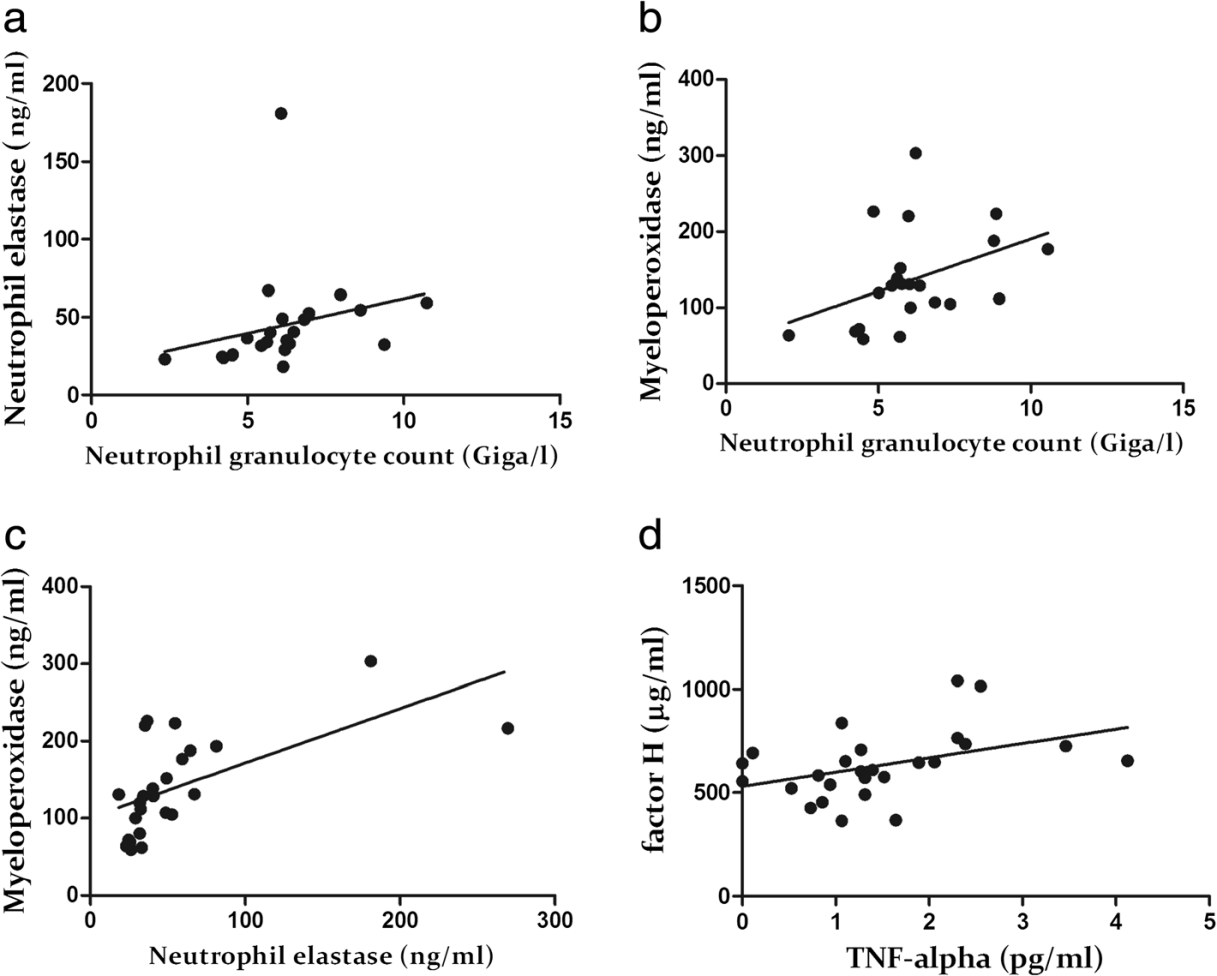
Statistically significant correlations between neutrophil granulocyte count and neutrophil elastase level (**a**), neutrophil granulocyte count and myeloperoxidase level (**b**), neutrophil elastase and myeloperoxidase levels (**c**) and factor H and TNF-α levels (**d**) measured in the samples drawn during attacks. Spearman’s rank correlation coefficient was calculated

On the other hand, we found many relationships among the indices determined in the blood samples drawn during edematous episodes. There was a strong positive correlation between NGC and NE level (*R* = 0.6512, *p* = 0.0008), and a slightly positive correlation between NGC and MPO level (*R* = 0.4241, *p* = 0.0492). An even closer relationship was revealed between NE and MPO levels (*R* = 0.7090, *p* < 0.0001). Moreover, we found a positive correlation also between factor H and TNF-α levels (*R* = 0.5061, *p* = 0.0083) (Fig. 4). Significant relationships could not be found for CRP, IL-8 and C5a.

Further, we searched for possible relationships among the measured markers of neutrophil activation and the diagnostic parameters of C1-INH-HAE (antigenic and functional C1-INH, as well as C4 levels). None of these parameters exhibited a significant correlation –neither in samples from symptom-free periods, nor in those obtained during edematous episodes.

## Discussion

In our current study, we confirmed that NGC indeed increases during edematous episodes of C1-INH-HAE—even if the effect of hemoconcentration is taken into account. Our study showed for the first time that NGC is higher in C1-INH-HAE patients during symptom-free period than in healthy controls. Further studies are necessary to elucidate the mechanism behind this elevation and its possible relationship with C1-INH deficiency. We found that NGs undergo activation during edematous episodes. This is evidenced by the elevated levels of NE, MPO, and PTX3 (all released from the granules of the NGs) relative to those found in blood samples obtained during symptom-free periods from the same patients. Apparently, the activation of NGs is indeed related to edema formation. In particular, the elevated NGC found in symptom-free patients is not accompanied by an enhanced release of granulocytic enzymes, when compared with the values measured in healthy controls.

In addition to elevated NE and MPO levels, the occurrence of neutrophil activation during edematous episodes is suggested also by the positive correlation between NGC and NE level. This can be observed only in blood samples drawn during edematous attacks, but not in those obtained during symptom-free periods from the same patients, or from healthy individuals. The relationship between the levels of NE and of MPO exhibited an even stronger correlation. These relationships and the elevation of PTX3 level during the attacks might suggest the expression of NETs.

TNF-α and IL-8 are possible activators of neutrophil functions [21–23]. We found elevated levels of these factors in blood samples drawn during edematous attacks, compared with those from symptom-free periods. Remarkably, TNF-α levels were lower in the samples of symptom-free patients than in those from healthy controls. Although we confirmed this finding by extending the measurements to samples from additional patients and controls, we cannot offer any ideas as to its significance. Only limited data have been published on the role of cytokines in C1-INH-HAE, and even these are available from a small number of cases. Most of these studies did not compare samples obtained from the same patients during and between edematous attacks, and all analyzed serum samples [24–26]. Serum is the least suitable sample type for measuring cytokine levels. According to comparative appraisals, EDTA plasma is the most appropriate for this purpose, because it is more stable than lithium-heparin, ammonium-heparin or, serum samples [27, 28].

The elevated level of TNF-α and IL-8 seen during edematous episodes might be related to neutrophil activation. On one hand, these cytokines could activate NGs and on the other hand, the latter themselves can produce both cytokine [20].

We made the interesting observation that serum CRP level was elevated in the symptom-free period, compared with that observed in the healthy subjects, and it increased further significantly during attacks in the same C1-INH-HAE patients. The latter observation is in agreement with previous findings [29]. Although NGs are inflammatory cells, we could not detect any significant correlation among NGC, TNF-alpha, and CRP levels.

Complement is a first-line defense of innate immunity, which aids the clearance of pathogens by opsonic, lytic, inflammatory, and immunomodulatory activities [30]. The C5a complement anaphylatoxin is a strong chemoattractant for neutrophils and a mediator of neutrophil adhesion [31, 32]. Factor H is a complement inhibitor, and also controls cell activation and adhesion through binding to the iC3b receptors present on the surface of neutrophils [19]. We did not find any differences between C5a and factor H levels measured in samples from symptomatic or symptom-free patients, or from healthy controls. Therefore, it appears that these complement factors are unlikely to be responsible for the neutrophil activation occurring during edematous attacks.

## Conclusion

The activity of systemic mechanisms during edematous episodes suggests their role in edema-formation, even though the latter is a localized process in C1-INH-HAE. Our study confirmed that NGC increases during edematous attacks in patients with C1-INH-HAE, and pointed out that these cells undergo activation. However, questions arise about the cause behind the increase in the numbers and the activation of NGs and it needs further studies to determine whether NG activation is a cause or a consequence of an edematous attack. The role of these cells in the spontaneous resolution of edema is similarly unclear.

As it might prove a new, important aspect of the pathomechanism of C1-INH-HAE, we plan further research into the circumstances of, as well as the causality behind neutrophil activation.

## Abbreviations

C1-INH-HAE: Hereditary angioedema due to C1-inhibitor deficiency
C1-INH: C1-inhibitor
HK: High molecular weight kininogen
NE: Neutrophil elastase
NG: Neutrophil granulocyte
NGC: Neutrophil granulocyte count
MPO: Myeloperoxidase
PTX3: Pentraxin 3
IL: Interleukin
TNF-α: Tumor necrosis factor-α
WBC: White blood cell
RBC: Red blood cell
NET: Neutrophil extracellular trap
PK: Prekallikrein
